# An Integrated Analysis of the Rice Transcriptome and Metabolome Reveals Root Growth Regulation Mechanisms in Response to Nitrogen Availability

**DOI:** 10.3390/ijms20235893

**Published:** 2019-11-24

**Authors:** Wei Xin, Lina Zhang, Wenzhong Zhang, Jiping Gao, Jun Yi, Xiaoxi Zhen, Ming Du, Yanze Zhao, Liqiang Chen

**Affiliations:** 1 Key Laboratory of Northern Japonica Rice Genetics and Breeding, Ministry of Education and Liaoning Province, Key Laboratory of Northeast Rice Biology and Genetics and Breeding, Ministry of Agriculture, Rice Research Institute of Shenyang Agricultural University, Shenyang 110866, China; xinwei2017@stu.syau.edu.cn (W.X.); yijun89@syau.edu.cn (J.Y.); xiaoxizhen1991@163.com (X.Z.); lestat1122@126.com (M.D.); zyz13352469165@163.com (Y.Z.); syndtonyqiang@163.com (L.C.); 2 Graduate School of Agricultural Science, Tohoku University, Sendai 981-8555, Japan; zhanglina921210@gmail.com

**Keywords:** rice, transcriptome, metabolome, root architecture

## Abstract

Nitrogen is an essential nutrient for plant growth and basic metabolic processes. Root systems play an important role in the ability of plants to obtain nutrients from the soil, and are closely related to the growth and development of above-ground plants. Root morphology analysis showed that root growth was induced under low-nitrogen conditions and inhibited under high-nitrogen conditions. To better understand the molecular mechanisms and metabolic basis underlying the rice root response to nitrogen availability, an integrated analysis of the rice root transcriptome and metabolome under three environmental conditions (low-, control, and high-nitrogen conditions) was conducted. A total of 262 and 262 differentially level metabolites were identified under low- and high-nitrogen conditions, respectively. A total of 696 and 808 differentially expressed genes were identified under low- and high-nitrogen conditions, respectively. For both the differentially expressed genes and metabolites, KEGG pathway analysis indicated that amino acid metabolism, carbon and nitrogen metabolism, phenylpropanoid metabolism, and phytohormones’ signal transduction were significantly affected by nitrogen availability. Additionally, variable levels of 65 transcription factors (TFs) were identified in rice leaves exposed to high and low nitrogen, covering 22 TF families. These results also indicate that there is a significant difference in the transcriptional regulation mechanisms of rice roots between low and high nitrogen. In summary, our study provides new information for a further understanding of the response of rice roots to low-nitrogen and high-nitrogen conditions.

## 1. Introduction

Rice is one of the most widely grown food crops globally, and approximately 50% of the global population eat rice as a staple food [1]. The sustainable development of rice production is an important aspect of guaranteeing food security. In recent decades, rice yields have continued to increase due to the application of a large number of chemical fertilizers, particularly nitrogen [2,3]. However, with this significant increase in rice yield, considerable and unreasonable nitrogen fertilizer application has also caused many problems such as the reduction of the nitrogen fertilizer utilization rate, a rise in production costs, and environmental pollution, which have seriously influenced the sustainable development of rice production [4,5]. Therefore, ways must be found to enhance rice production and nitrogen use efficiency (NUE) in order to meet the growing needs of the population and reduce environmental costs.

Roots are essential for plant growth and development, anchoring plants to growth substrates, promoting the uptake of water and nutrients from the soil, and responding to biotic and abiotic stresses [6]. Root architectural and physiological characteristics are closely related to the growth of the shoot, yield formation, and nitrogen uptake and utilization. Fan et al. [7] found that rice root architecture characteristics and physiological activity have significant effects on nitrogen use efficiency at various stages of rice growth. Nitrogen is also a mineral nutrient that affects root architecture and physiological characteristics. Previous studies have shown that the nitrogen nutrient supply level has a serious impact on rice root architectural and physiological characteristics [3]. Francisco et al. [8] showed that appropriate nitrogen deficiency promotes root growth and facilitates deep rooting. WALCH-LIU [9] found that excessive nitrogen application does not continue to increase the number of adventitious roots, while reducing root morphological parameters and ultimately inhibiting root growth. Therefore, understanding the molecular mechanisms that control root development is critical to improving nutrient uptake efficiency and yield in crops.

Nutrient management, as an important means of cultivation regulation, has become a popular topic in research on rice growth and development. In particular, root systems have been receiving increased research attention in recent years. At present, research on the effects of nitrogen on roots is more focused on the physiological aspects, and the related molecular regulation mechanisms remaining largely unknown. In recent years, with the development and application of high-throughput sequencing, high-resolution mass spectrometry, and information processing, systems biology (omics) research has become a focus point for exploring major scientific issues. The integrated analyses of transcriptomic and metabolomic data obtained from two biological levels, i.e., transcript and metabolite levels, are a useful way of examining the complexity of biological systems. The integrated analyses of transcriptomic and metabolomic data have been applied to the study of various aspects of plant biology, including the responses of *Arabidopsis thaliana* to nutritional stresses [10], the elucidation of gene-to-gene and metabolite-to-gene networks in *Arabidopsis* [11], the identification of *Fusarium* head blight resistance genes in wheat [12], rice insect interaction research [13], and duckweed responses to nitrogen starvation [14]. However, as far as we know, the research on the response of rice to nitrogen nutrition by an integrated analysis of the transcriptome and metabolome is scant.

In the current study, rice was exposed to low nitrogen, control nitrogen, and high nitrogen for 30 days. We measured the root architectural and physiological characteristics, as well as changes in transcription and metabolism among the three treatments. The integrated analysis of the transcriptome and metabolome allowed us to obtain more insight into the regulation of rice root architectural changes in response to nitrogen availability. The purpose of our study was to identify strategies rice roots use to respond to nitrogen availability, which could be used for research to improve nitrogen use efficiency (NUE) and yield in rice.

## 2. Results

### 2.1. Nitrogen Availability Affects Rice Root Architectural and Physiological Characteristics

**Figure 1:**
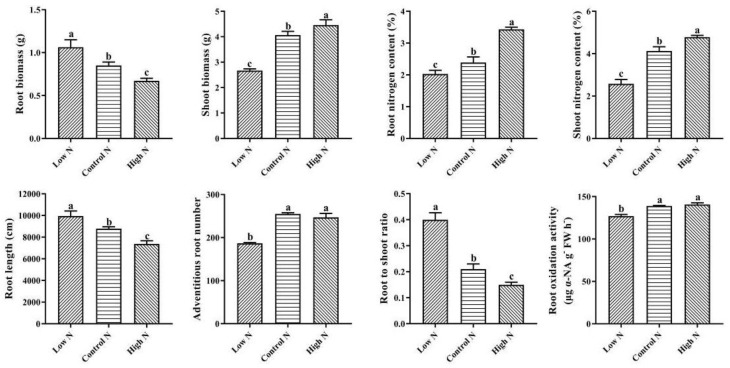
Rice root architectural and physiological characteristics’ response to low and high nitrogen. Values labeled with different letters in the same row indicate a significant difference between the nitrogen treatments (*p* values < 0.05, *n* = 3).

As shown in Figure 1, compared with control nitrogen, shoot biomass accumulation was inhibited by low nitrogen and promoted by high nitrogen, while root biomass accumulation was promoted by low nitrogen and inhibited by high nitrogen. Compared with control nitrogen, nitrogen deficiency had a negative effect on the root to shoot ratio, while high nitrogen increased the root to shoot ratio. The root length was significantly inhibited under high-nitrogen conditions compared with control nitrogen conditions, while root length was significantly promoted under low-nitrogen conditions. The shoot and root nitrogen content increased with increasing nitrogen supply levels. Compared with control nitrogen, low nitrogen decreased the adventitious root number and root oxidation activity, while high nitrogen had no significant effect on adventitious root number and root oxidation activity.

### 2.2. Metabolite Profiles of Rice Roots in Response to Nitrogen Availability

**Figure 2:**
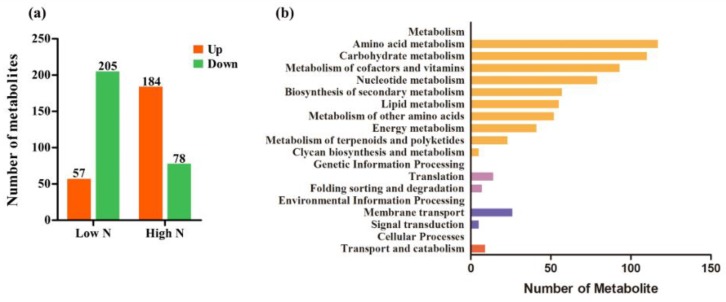
Metabolic profile analysis of rice roots under low- and high-nitrogen conditions: (**a**) the total number of metabolites with differential levels, upregulated and downregulated, under different nitrogen treatments; (**b**) KEGG pathway enrichment analysis of these changed metabolites.

In order to obtain an overview of metabolic changes in response to nitrogen availability, nontargeted metabolic analysis was performed using LC-ESI-MS/MS. As shown in Figure 2a, compared with control nitrogen, a total of 351 metabolites were determined as having differential levels under low and high nitrogen. Among them, 262 metabolites’ levels changed under the low nitrogen condition: 205 metabolites’ levels decreased and 57 metabolites’ levels increased. A total of 262 metabolites changed under high-nitrogen conditions: 78 metabolites’ levels decreased and 184 metabolites’ levels increased. The increased levels of metabolites were higher than the decreased levels of metabolites under low nitrogen, while the opposite was the case under high nitrogen. Root architecture analysis showed that root growth was promoted by low nitrogen and inhibited by high nitrogen. These results indicated that changes in root metabolite levels are closely related to root growth. KEGG metabolic pathway enrichment analysis classified the differential metabolites identified under low and high nitrogen into metabolism, genetic information processing, environmental information processing, and cellular processes (Figure 2b). Within metabolism, the enriched pathways were amino acid metabolism, carbohydrate metabolism, and metabolism of cofactors and vitamins. Within genetic information processing, the enriched pathways were translation and folding, sorting, and degradation. Within environmental information processing, the enriched pathways were membrane transport and signal transduction. Within cellular processes, the enriched pathways were transport and catabolism. These results indicated that the metabolic progress of rice roots was significantly affected by nitrogen nutrient supply.

**Figure 3:**
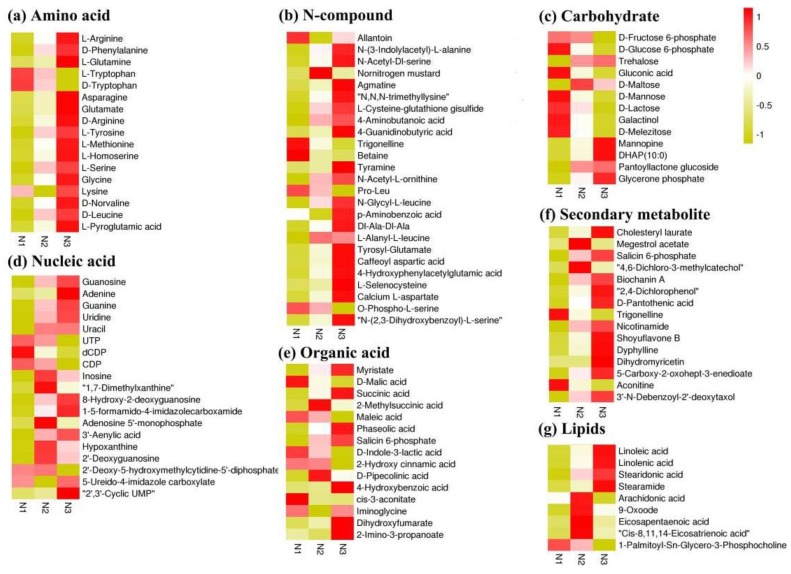
Heat map of differentially changed metabolites under low- and high-nitrogen conditions. N1, N2, and N3 indicate metabolite levels of low nitrogen, control nitrogen, and high nitrogen, respectively. (**a**) Amino acid; (**b**) N-c0mpound; (**c**) Carbohydrate; (**d**) Nucleic acid; (**e**) Organic acid; (**f**) Secondary metabolite; (**g**) Lipids.

Annotated analysis of integrated KEGG, HMDB, and METLIN databases showed that amino acids, nitrogenous compounds, carbohydrates, nucleic acids, organic acids, fatty acids, and secondary metabolites were significantly different for different nitrogen supply levels (Figure 3). In addition, 17 amino acids were determined; with the exception of l-Tryptophan, d-Tryptophan, and Lysine, all increased under high nitrogen and decreased under low nitrogen. l-Tryptophan and d-Tryptophan increased under low nitrogen and decreased under high nitrogen. Most N-compounds were increased by high nitrogen and decreased by low nitrogen; among these, allantoin, trigonelline, betaine, pro-leu, and O-phospho-l-serine were increased by low nitrogen compared with control nitrogen. The levels of most of the differentially changed metabolites involved in nucleic acids and secondary metabolites were increased under high-nitrogen conditions, while some carbohydrates showed an increased level under low-nitrogen conditions.

### 2.3. RNA Sequencing Profiles of Rice Roots in Response to Nitrogen Availability

**Figure 4:**
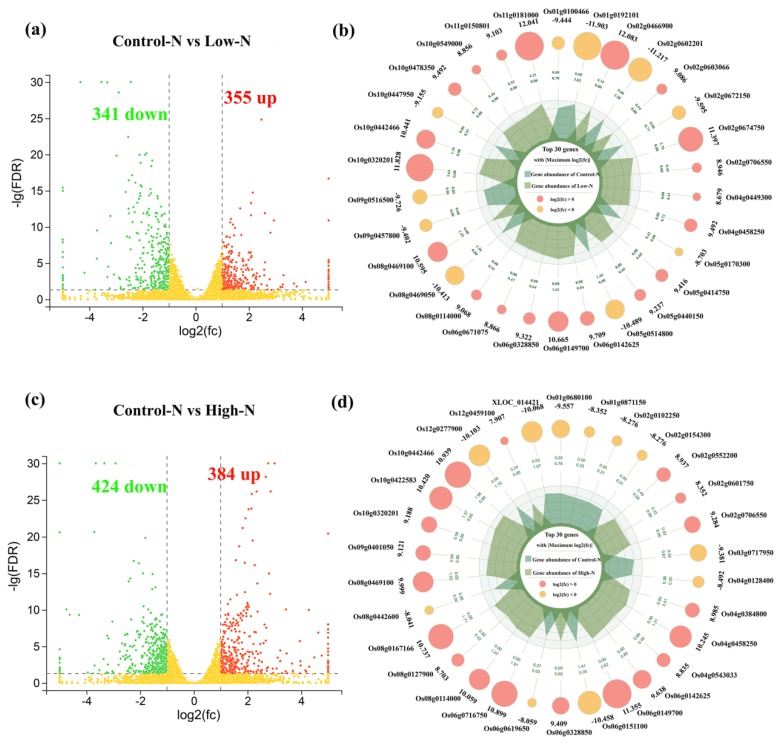
Transcriptional analysis of rice roots under low- and high-nitrogen conditions. (**a**) The number of DEGs and (**b**) top 30 DEGs under low-nitrogen conditions. (**c**) The number of DEGs and (**d**) the top 30 DEGs under high-nitrogen conditions. The top 30 DEGs were selected based on |log2FC|. −lg(FDR), −log10 (false discovery rate), FC, fold change.

The analysis of differential gene expression was performed using edgeR software under low- and high-nitrogen conditions, and the results are shown in Figure 4. DEGs were selected by FDR and log2FC with thresholds of FDR < 0.05 and |log2FC| > 1. Compared to the control treatment, 696 genes were differentially expressed under low-nitrogen conditions, with 355 upregulated and 341 downregulated. Under high-nitrogen conditions, 808 genes were differentially expressed, with 384 upregulated and 424 downregulated. The top 30 DEGs were selected based on |log2FC|. Notably, nine DEGs exhibited regulatory responses to both high- and low-nitrogen conditions. According to functional annotations of their reference genes (Ensembl release 38 IRGSP-1.0), two candidates (*OsCYSK* and *OsNEP1*) were involved in amino acid metabolism and energy metabolism. Meanwhile, seven candidate genes had no functional annotations and an intensive response to nitrogen availability. Although they had no functional annotations, we still consider their specific importance in rice roots responding to nitrogen availability changes. Transcriptome profiles were confirmed using qRT-PCR. The expression patterns of 27 randomly selected genes were similar to those obtained from RNA-Seq analysis the Pearson coefficient was 0.91 (Figure S1), confirming the reliability of the RNA-Seq data.

To further understand the functions of the DEGs and the related biological processes they participate in, GO and KEGG enrichment analyses were conducted. Using GO analysis, DEGs under low and high nitrogen were classified into cellular component, molecular function, and biological process, involving 28 GO terms (Figure S2). Within the cellular component, the enriched GO terms were cell, cell part, and organelle. Within molecular function, the enriched GO terms were catalytic activity, binding, and transporter activity. Within biological process, the enriched GO terms were metabolic processes, cellular processes, and single-organism processes. Pathway enrichment analysis of the DEGs identified in the present study using KEGG identified the terms carbohydrate metabolism, energy metabolism, amino acid metabolism, biosynthesis of secondary metabolites, signal transduction, and lipid metabolism as being significantly enriched in these genes compared against the whole genomic background (Figure S3). The expression of most amino acid metabolism-related genes was reduced under low-and high-nitrogen conditions. The expression of most carbohydrate metabolism-related genes was reduced under low-nitrogen conditions, and increased under high-nitrogen conditions. Meanwhile, the expression of genes associated with ‘energy metabolism’ was inhibited under low-nitrogen conditions.

### 2.4. Regulation of Differential Gene Expression by Transcription Factors

Transcription factors can regulate the expression of other genes and play an important role in regulating plant growth and adapting to biotic and abiotic stresses [15]. As shown in Table 1, a total of 65 TFs were identified in rice leaves exposed to high and low nitrogen, covering 22 TF families (Table S1). Compared to the control nitrogen conditions, 48 TFs were differentially expressed under low-nitrogen conditions, with 35 upregulated and 13 downregulated. Under high-nitrogen conditions, 23 TFs were differentially expressed, with 10 upregulated and 13 downregulated. Overall, most TFs were upregulated under low nitrogen and unchanged or downregulated under high nitrogen. The bHLH, NAC, MYB-related, WRKY, and ERF families are relatively large TF families responsive to nitrogen availability. Although the MYB-related and NAC family members were responsive to different nitrogen supply levels, four MYB-related were detected in low nitrogen, three of which were upregulated, whereas six MYB-related family TFs detected under high-nitrogen conditions were downregulated. Six NAC TFs were detected in low nitrogen, five of which were upregulated, while three NAC family TFs detected under high-nitrogen conditions were downregulated. C2H2 and WRKY family members were specifically upregulated under low-nitrogen conditions.

### 2.5. Phenylpropanoid Metabolism Modulated by Nitrogen Availability

**Figure 5:**
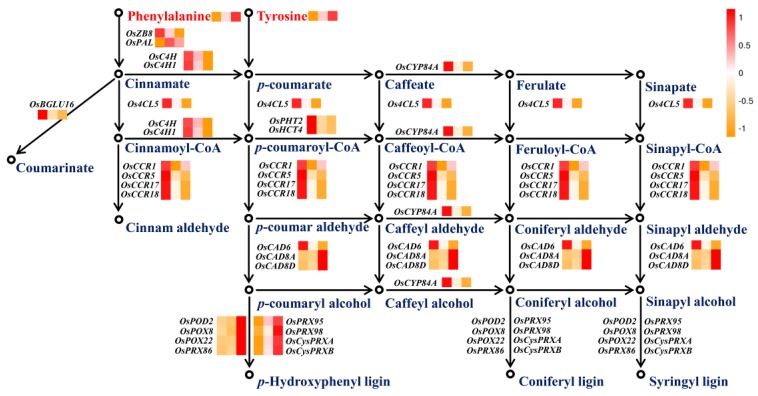
Metabolites and transcripts enriched in phenylpropanoid biosynthetic pathways under low- and high-nitrogen conditions. Three squares under the metabolite or transcript names indicate the changed level or expression abundance under low-, control, and high-nitrogen conditions.

An integrated analysis of KEGG pathway enrichment was performed between the transcriptome and metabolome. Three pathways were enriched under high-nitrogen conditions, including phenylalanine metabolism, isoquinoline alkaloid biosynthesis, and nitrogen metabolism (Figure S4a). Correspondingly, three pathways were also enriched under low-nitrogen conditions, namely, nitrogen metabolism, arginine biosynthesis, and biosynthesis of amino acids (Figure S4b). These results suggest that the metabolism of nitrogen, amino acids and, in particular, phenylalanine are important in regulating root architecture and physiological characteristics under both high and low nitrogen. As shown in Figure 5, phenylalanine and tyrosine were increased under high-nitrogen conditions, and decreased under low-nitrogen conditions. The abundance of expressed phenylpropanoid biosynthesis genes *OsPAL7*, *Os4CL5*, *OsCCR1*, *OsCCR5*, *OsCCR17*, *OsCCR18*, *OsC4H*, *OsC4H1*, *OsPHT2*, *OsHCT4*, *OsCYP84A*, and *OsBGLU16* increased under low-nitrogen conditions, and were all inhibited by high nitrogen with the exception of *OsCCR1*. The abundance of the cinnamyl-alcohol dehydrogenase encoded gene product of *OsCAD6* increased under both low- and high-nitrogen conditions. The expression of *OsCAD8A* and *OsCAD8D* was increased by high nitrogen, but unchanged under low-nitrogen conditions. A total of eight peroxidases involved in phenylpropanoid biosynthesis were identified as DEGs under low- and high-nitrogen conditions. All genes were upregulated under high-nitrogen conditions; among them, four genes were downregulated under low-nitrogen conditions.

### 2.6. Nitrogen Availability Affects the Levels of Phytohormones Related to Growth and Stress Responses

**Figure 6:**
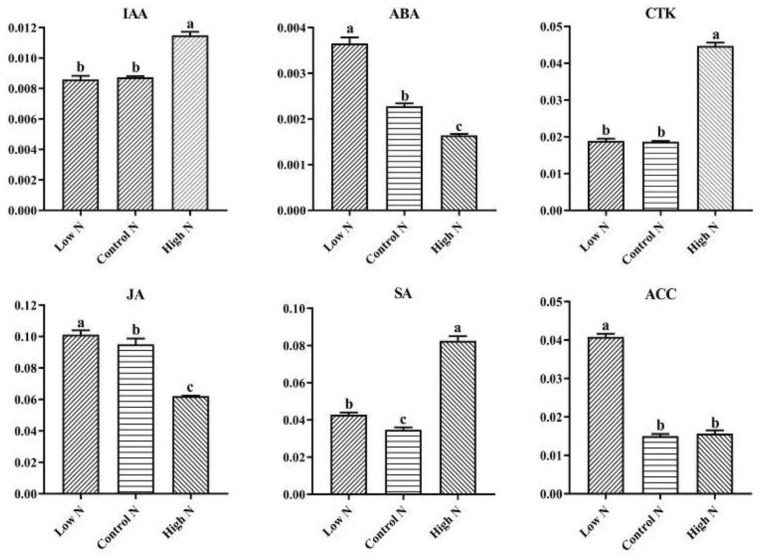
Concentrations (ng g^−FW^) of IAA, ABA, CTK, JA, SA, and ACC in the roots of rice exposed to low-, control, and high-nitrogen conditions. Values labeled with different letters in same row indicate significant difference between the nitrogen treatments (*p* values < 0.05, *n* = 3).

Plant hormones play an important role in plant growth and adaptation to biotic and abiotic stresses, as well as sensing changes in environmental conditions, especially nutrient availability. Indeed, the results of KEGG enrichment analysis of DEGs showed that the plant hormone signal transduction metabolic pathway was significantly enriched under low- and high-nitrogen conditions (Table S2). Therefore, we analyzed changes in the concentrations of IAA, ABA, CTK, JA, SA, and ACC in the roots of rice when treated with either low nitrogen or high nitrogen (Figure 6). Compared with the control nitrogen conditions, IAA and CTK content showed no significant changes, while ABA, JA, SA, and ACC content were increased under low-nitrogen conditions. IAA, CTK, and SA were increased, ABA and JA were decreased, and ACC showed no significant changes under high-nitrogen conditions.

## 3. Discussion

Roots play an important role in connecting the plant to the soil and thus the soil to the atmosphere. The growth of above-ground plants is determined by the availability of nutrients and water, and is therefore closely related to root architecture and physiology [7,16,17]. In this study, nitrogen deprivation induces root biomass and root length, and inhibits adventitious root number, while excess nitrogen inhibits root biomass and root length. ROA was considered to be an important index of root physiological activity (Figure 1). The present results showed that ROA was significantly inhibited by low nitrogen [18,19]. These results suggested that the rice root architecture and physiology showed significant changes under low- and high-nitrogen conditions. However, the adaptation mechanisms for nitrogen availability were not exactly the same. To further clarify the genetic and metabolic basis of nitrogen regulation of root growth and development, we used integrated analysis of the transcriptome and metabolome. KEGG pathway analysis indicated that amino acid metabolism, carbon and nitrogen metabolism, phenylpropanoid metabolism, and phytohormones’ signal transduction were significantly affected by nitrogen availability (Figure S3). Compared with our previous studies on the response of above-ground plants to both low nitrogen and high nitrogen, we found that phytohormones’ signal transduction was significantly affected in rice roots [20]. This may be related to the roots being in directly contact with the external environment under both low- and high-nitrogen conditions. Inorganic nitrogen is absorbed and transported by specific transport proteins, such as ammonium transporters (AMTs) and nitrate transporters (NRTs) [21]. We found that *OsNRT2.4* was upregulated under low nitrogen and downregulated under high nitrogen (Figure S3). Furthermore, in the current study, nitrogen assimilation-related DEGs were significantly downregulated under low nitrogen, and no significant difference was found under high nitrogen (Figure S3). Interestingly, we found that rice root amino acids increased with increasing nitrogen levels, while carbohydrate changes were not consistent with amino acid changes (Figure 3). Phenotypic analysis also showed that the rice root to shoot ratio decreased with increasing nitrogen levels (Figure 1). These results indicated that, under low-nitrogen conditions, compensatory growth of rice roots occurred through the coordination of carbon and nitrogen metabolism to maintain normal growth.

The phenylpropanoid metabolic pathway is closely related to the synthesis of lignin [22]. Lignin is the main component of the plant skeleton, and plays an important role in the plant roots’ growth [23]. Phenylalanine ammonia-lyase (PAL), 4-coumarate-CoA ligase (4CL) cinnamyl-alcohol dehydrogenase (CAD), and cinnamoyl-CoA reductase (CCR) are the main enzymes involved in the lignin-specific synthesis pathway [24]. The expression site of *OsCCR1* was concentrated in the area with a high degree of lignification and is directly involved in lignin synthesis [25]. In this study, we identified that most of the phenylpropanoid biosynthesis-related genes were upregulated under low nitrogen and downregulated under high nitrogen (Figure 5). Interestingly, under low-nitrogen conditions, peroxidase-encoding genes involved in phenylpropanoid biosynthesis showed reduced expression levels, while their expression levels were increased under high nitrogen (Figure 5), which is consistent with previous results in maize [26], rapeseed [27], and rice [20]. According to previous studies, oxygen free radicals and hydrogen peroxide are the two main reactive oxygen species that are differentially distributed in *Arabidopsis* roots [28]. Qin et al. [26] considered that decreased abundance of peroxidases proteins may promote rapeseed root growth by reducing apoplastic hydrogen peroxide levels and the production of oxygen radicals to destroy cell wall structures, consequently stimulating the cell wall loosening process and root growth. Raggi et al. [29] found that *AtPRX71* in *Arabidopsis* is closely associated with cell expansion. In addition, endogenous β-glucanase (EGase), a key enzyme of phenylpropanoid biosynthesis, plays an important role in the growth and development of plants. The EGase-associated genes were isolated from the apical yellowing seedling hypocotyls of peas, the elongation of the inflorescence of *Arabidopsis thaliana*, and the elongation of tobacco plants and roots. There is high expression of EGase in the extended region of the plant, but it is unexpressed or downregulated in the region where extension has stopped, indicating that EGase plays an important role in the elongation of plant cells [30,31,32] and may be directly involved in the biosynthesis of cellulose in tissues [33]. In this study, the expression of the EGase-encoding gene (*OsBGLU16*) was significantly upregulated under low-nitrogen conditions (Figure 5). These results indicate that differences in phenylpropanoid metabolic pathways are the main factors causing differences in root morphology between low nitrogen and high nitrogen.

Plants undergo a series of physiological, molecular, and developmental changes in response to nitrogen availability in the environment. Previous studies have shown that nitrogen transporters, assimilation enzymes, and signaling pathways are regulated by transcription factor in response to nitrogen availability [34,35]. Calcium and phosphorylation-dependent signaling cascades are also key regulators of this transcriptional response. Previously, 16 transcription factors that play an important role in nitrogen metabolism have been identified in *Arabidopsis*, but only seven have a demonstrated role in the regulation of root development in a nitrogen-dependent manner. Yang et al. [5], using RNA-Seq analysis, detected 85 TFs that play an important role in responding to nitrogen stress and regulating plant growth. In the current study, TF families such as bHLH, NAC, MYB-related, WRKY, and ERF were identified as being responsive to nitrogen availability, of which the WRKY family’s members were specifically upregulated under low-nitrogen conditions (Table 1). Heerah et al. [36] found that WRKY1 activates the transcription of *GDH1*, *NIA1*, *NIA2*, *NRT2.1*, and *AMT1.1* in *Arabidopsis*. Imamura et al. [37] also found that in *Cyanidioschyzon merolae* MYB1 can enhance nitrogen assimilation in nitrogen deficiency by regulating NRT, NAR, NIR, and GS expression. In addition, we found that most TFs were upregulated under low-nitrogen conditions, and experienced no change or were downregulated under high-nitrogen conditions (Table S1). These results show that there is a significant difference in the transcriptional regulation mechanisms of rice roots between low nitrogen and high nitrogen. In future studies, it will be important to determine the role of nitrogen-responsive transcription factors for increasing rice yield and reducing fertilizer overuse.

Plant hormones play an important role in regulating the growth and development of higher plants. The synthesis and action of plant hormones are affected by environmental factors, such as the supply of mineral nutrients. In the current study, according to our transcriptome and data on several phytohormones’ content, the expression of genes involved in signal transduction pathways associated with several plant hormones, and several phytohormones’ content, were altered after nitrogen treatment for 30 days (Table S2), suggesting that hormone pathways play critical roles in the rice root response to nitrogen availability. Previous studies have also demonstrated that nitrogen and hormonal signals can jointly regulate plant morphological and physiological changes in response to nitrogen availability [38,39], although the general trend of CTK and IAA is to promote the growth and development of plants. In the current study, compared with normal nitrogen, the IAA and CTK contents did not change significantly under low nitrogen, and significantly increased under high nitrogen (Figure 6). Transcriptome analysis revealed that purine catabolism and zeatin biosynthesis, associated with CTK synthesis, were also significantly affected by nitrogen availability (Table S2). These results suggest that excess nitrogen caused the levels of IAA and CTK in rice roots to be higher than appropriate, and suppressed rice root growth under high nitrogen. The research of Krouk et al. [40] showed that the nitrate transporter NRT1.1 also transports IAA in *Arabidopsis* roots. ABA is considered a necessary messenger in the plant adaptive response to abiotic stresses. In response to environmental stresses, the level of endogenous ABA rapidly increases, which in turn activates specific signaling pathways and modifies gene expression [41,42]. In the current study, we found that ABA was inhibited by high nitrogen and promoted by low nitrogen (Figure 6). Furthermore, we detected decreased expression of two *PP2Cs* (Os03g0268600, Os05g0457200) under high nitrogen, and increased expression of two *PP2Cs* (Os03g0268600, Os09g0325700) under low nitrogen (Table S2), which are key components in the ABA signaling pathway, [43,44]. These results highlight the role of ABA signaling in fine-tuning nitrogen acquisition and root architecture by rice roots in response to nitrogen availability. Kiba et al. [45] showed that ABA is also involved in N-regulated root growth and nitrogen acquisition, although whether JA, SA, and ACC are involved in plant nitrogen signaling and metabolism is currently unknown [38]. Changes in the content of JA, SA, and ACC and expression levels of JA-, SA-, and ACC-mediated genes in rice roots after nitrogen treatment (Figure 6, Table S2) indicate that these phytohormones also play important roles in responding to changes in the nitrogen availability and in regulating the growth of rice roots.

In this study, these results provide a global view of architectural changes, and the underlying genetic and metabolic basis of nitrogen regulation of root growth and development. Changes in the expression of phenylpropanoid biosynthesis-related genes alter the rice root architecture in response to nitrogen availability. Phytohormones and transcription factors play an important role in adapting to low- and high-nitrogen conditions, as well as regulating root architecture changes.

## 4. Materials and Methods

### 4.1. Plant Material and Growth Conditions

The experiment was conducted in 2018 at the Rice Experiment Base of Shenyang Agricultural University, Liaoning Province, China (41°49′25″N, 123°34′15″E) during the rice growing season. After germination on moist filter paper, rice (*Oryza sativa L.*) seeds (cultivars, cv. “Shennong265” *Japonica* China) were disinfected with 0.01% HgCl_2_ for germination. Two days later, seedlings were cultured in a greenhouse (28/25 °C, 10 h day/14 h night). At the three-leaf heart stage, the seedlings were transferred to low nitrogen (13.33 ppm), control nitrogen (40 ppm), or high nitrogen (120 ppm) using NH_4_NO_3_ as the N source, and grown under natural conditions for 30 days. The hydroponics solution was formulated according to the method of Li et al. [46], and amended by adding 1 mL of dicyanamide (nitrate inhibitor) per 1 L of nutrient solution.

### 4.2. Analysis of Root Architecture and Physiological Characteristics

After 30 days of nitrogen treatment, the root architecture and physiological characteristics were investigated. Roots were scanned (Epson Expression 1680 Scanner, Seiko Epson Corp., Tokyo, Japan) and the adventitious root number and root length measured with WinRHIZO Pro 2013e software (Regent Instruments Inc., Quebec, Canada). Then, the shoots and roots were dried in an oven at 80 °C to a constant weight for measuring the root biomass and calculating the root to shoot ratio. Nitrogen content was measured with an elemental analyzer (Elementar Vario MACRO cube, Hanau, Germany). Root oxidation activity was determined according to the method of Ramasamy et al. [47]. Statistical tests were performed with SPSS (version 19) statistical software and visualizations were drawn using GraphPad Prism (version 8) software. Data were tested to confirm their normality before statistical analyses. For experimental variables, one-way ANOVA was applied to assess differences among treatments. Significant differences (*p* < 0.05) between treatments were indicated by different letters according to the ANOVA *F*-test.

### 4.3. Metabolite Extraction and Liquid Chromatography Electrospray Ionization Tandem Mass Spectrometry (LC-ESI-MS/MS) Analysis

Rice roots were obtained from three nitrogen treatments for 30 days; six biological replicates per treatment were collected for metabolic analyses. Metabolic extraction was based on the method of Xu et al. [48] LC-ESI-MS/MS analysis consisted of an ultrahigh-performance liquid chromatography system (UHPLC Agilent 1290, Santa Clara, CA, USA) fitted with a high-resolution mass spectrometer (Q Exactive Orbitrap, city, state, USA) equipped with an ESI interface. An ACQUITY UPLC HSS T3 column (1.7 μm × 2.1 mm × 100 mm, Waters) was used for chromatographic separation. The sample injection volume was 1 μL. Spectra were obtained using positive ion mode (POS) and negative ion mode (NEG). The ESI ion source spray voltage was 3800 V (POS) or −3100 V (NEG). The capillary temperature was maintained at 320 °C. Sheath gas and auxiliary gas flow rates were 45 and 15 Arb, respectively. The scan range was from 70 to 1000 *m*/*z* with primary resolution of 70,000 and secondary resolution of 17,500. Metabolites analyses were performed with ProteoWizard (version 3.0.6839) and XCMS (version 1.22.01) software. Metabolites were identified according to self-built databases (OSI-SMMS software) and public databases (HMDB, METLIN, KEGG) [49,50]. Differential metabolites were screened with VIP and *p* values as thresholds under three treatments (VIP ≥ 1, *p* < 0.05).

### 4.4. RNA Extraction and Sequencing

The total RNA of rice roots was extracted from three biological replicates with TRIzol reagent (Thermo Fisher Scientific, Waltham, MA, USA), according to the manufacturer’s protocol. The mRNA was enriched using Oligo (dT) beads; then the purified mRNA was fragmented into short fragments using fragmentation buffer and reverse-transcribed into cDNA by random hexamer primers. Second-strand cDNAs were synthesized using DNA polymerase I, RNase H, dNTP, and buffer. Then the cDNA fragments were purified using a QiaQuick PCR extraction kit, end repaired, poly (A) was added, and the fragments were ligated to Illumina (Guangzhou, China) sequencing adapters. The ligation products were size selected by 1% agarose gel electrophoresis, PCR amplified, and sequenced using Illumina HiSeqTM 2500. Filtering of clean reads: removing reads containing adapters; removing reads containing poly A bases; removing reads containing more than 10% of unknown nucleotides (N); removing low-quality reads containing more than 50% of low-quality (Q-value ≤ 20) bases. Alignment with ribosome RNA (rRNA): Short reads alignment tool Bowtie2 was used for mapping reads to the ribosome RNA (rRNA) database. The rRNA mapped reads will be removed. The remaining reads were further used in the assembly and analysis of transcriptome. Alignment with reference genome: The rRNA removed reads of each sample were then mapped to the reference genome by TopHat2. The alignment parameters were as follows: Maximum read mismatch is 2; distance between mate-pair reads is 50 bp; error of distance between mate-pair reads is ±80 bp. The reconstruction of transcripts was carried out with the software Cufflinks, which together with TopHat2 allows biologists to identify new genes and new splice variants of known ones. The program reference annotation-based transcripts (RABT) was preferred. Cufflinks constructed faux reads according to reference to make up for the influence of low coverage sequencing. During the last step of assembly, all of the reassembled fragments were aligned with reference genes and similar fragments were removed. Then we used Cuffmerge to merge transcripts from different replicas of a group into a comprehensive set of transcripts, and merged the transcripts from multiple groups into a final, comprehensive set of transcripts for further downstream differential expression analysis. Gene abundances were quantified by the software RSEM. There were two steps for RSEM to quantify gene abundances. First, a set of reference transcript sequences was generated and preprocessed according to known transcripts and new transcripts (in FASTA format) and gene annotation files (in GTF format). Secondly, RNA-seq reads were realigned to the reference transcripts by the Bowtie alignment program and the resulting alignments were used to estimate gene abundances. The gene expression level was normalized using the FPKM (fragments per kilobase of transcript per million mapped reads) method. Then, differential gene expression analysis between treatments was performed using edgeR software (Available online: http://www.r-project.org/) to screen DEGs (FDR < 0.05, |log2FC|>1) [51]. Genes were annotated against the Gene Ontology (GO) (Available online: http://www.r-project.org/) and Kyoto Encyclopedia of Genes and Genomes (KEGG) (Available online: http://kobas.cbi.pku.edu.cn/) databases to obtain their functions. Significant functional categories and metabolic pathways were identified within differentially expressed genes, with FDR ≤ 0.05.

### 4.5. Confirmation of Transcriptome Data Using qRT-PCR Analysis

The first-strand cDNA was synthesized using the Prime Script RT Master Mix (Takara, Tokyo, Japan). Real-time PCR was performed with SYBR Premix Ex Taq II (Takara, Tokyo, Japan) according to the manufacturer’s protocol in Applied Biosystems QuantStudio 3 (Thermo Fisher Scientific, Waltham, MA, USA); transcript-specific primers are listed in Table S3. Relative quantification analysis was performed with a relative standard curve for threshold values (CT). qRT-PCR data were standardized using OsACTIN1 as an internal reference. Correlation coefficients for RNA-Seq and qRT-PCR data were plotted and calculated using Origin (version 9) mapping software.

### 4.6. Determination of Phytohormone Content

After grinding the samples in liquid nitrogen, a 50 ± 3 mg sample was placed in a 2-mL centrifuge tube, to which 50 μL internal standard solution and 1 mL acetonitrile aqueous solution (1% FA) were added. The sample was shaken and mixed for 2 min, stored at 4 °C in the dark for 12 h, and centrifuged at 14,000 g for 10 min. Then, 800 μL of supernatant was dried under a nitrogen stream and reconstituted with 100 μL of acetonitrile water (1:1, *v*/*v*). After centrifuging again at 14000× *g* for 10 min, the supernatant was taken for injection analysis. Samples were separated using a Waters I-Class LC ultrahigh-performance liquid chromatography system. In the mobile phase, the A solution was 0.05% FA aqueous solution, and the B solution is 0.05% FA acetonitrile. The sample was placed in a 4 °C autosampler at a column temperature of 45 °C with a flow rate of 400 μL/min and an injection volume of 2 μL. The relevant liquid phase gradient was as follows: 0–10 min, B liquid changes linearly from 2% to 98%; 10–10.1 min, B liquid changes linearly from 98% to 2%; 11.1–13 min, B liquid was maintained 2%. A QC book was set up for each experimental sample in the sample queue to detect and evaluate the stability and repeatability of the system. A QC sample was set for each experimental sample in the sample queue for the detection and evaluation system. Stability and repeatability. Mass spectrometry was performed in negative ion mode using a 5500 QTRAP mass spectrometer (AB SCIEX, Boston, MA, USA). The 5500QTRAP ESI was used to detect the pair of ions to be tested under the following conditions: source temperature 500 °C, ion Source Gas1: 45, Ion Source Gas2: 45, Curtain gas: 30, ionSapary Voltage Floating −4500 V; using MRM mode Detecting the pair of ions to be tested. The peak area and retention time were extracted using MultiQuant software. The amount of phytohormone measured in the sample was calculated based on the standard curve. 

## Figures and Tables

**Table 1:** Change in expression of transcription factors in rice roots in response to nitrogen availability.

TF Family	Low-N		High-N	
	Up	Down	Up	Down
bHLH	6	1	1	1
bZIP	1	1	1	1
C2H2	4	0	0	0
CO-like	1	0	1	0
DBB	0	2	0	0
E2F/DP	0	1	1	0
EIL	0	0	0	1
ERF	4	1	1	0
G2-like	0	1	1	0
GRAS	1	0	1	0
HD-ZIP	1	1	0	0
HSF	1	0	0	0
LSD	0	1	0	0
M-type_MADS	0	0	1	0
MYB	1	0	0	0
MYB_related	3	1	0	6
NAC	5	1	0	3
NF-YA	1	0	0	0
NF-YC	0	1	1	0
Nin-like	0	0	1	1
Whirly	0	1	0	0
WRKY	6	0	0	0
Total	35	13	10	13

## Supplementary Materials

Supplementary materials can be found at https://www.mdpi.com/1422-0067/20/23/5893/s1.
