# Supplementary material for: An Integrated Analysis of the Rice Transcriptome and Metabolome Reveals Root Growth Regulation Mechanisms in Response to Nitrogen Availability
Source: Int J Mol Sci. 2019 Nov 24;20(23):5893. doi: 10.3390/ijms20235893 (PMC6928638; doi:10.3390/ijms20235893)
Supplement: Supplementary file 1 [file ijms-20-05893-s001.pdf]

Supplementary Material

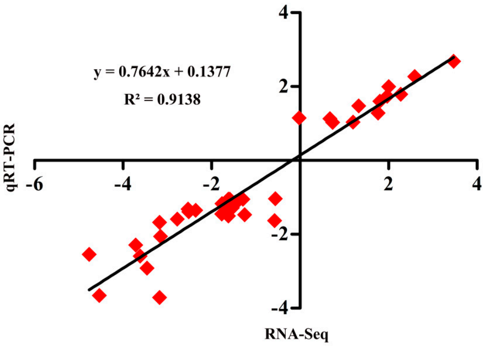

Supplementary Figure 1. A qRT-PCR assay was carried out for 27 randomly selected DEGs. Values are the $\log_2$ (FC) (low-N/control-N or high-N/control-N) for genes. The correlation coefficient (R^2) is indicated in the figure.

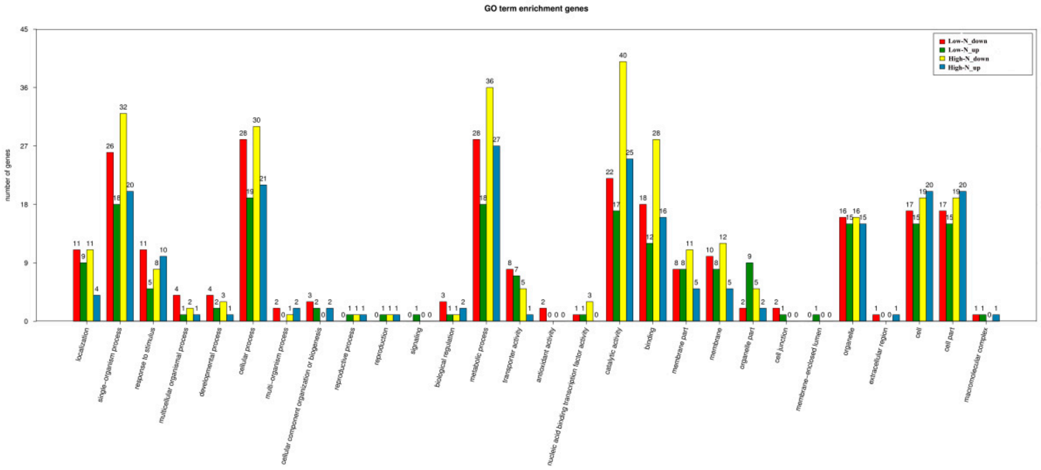

Supplementary Figure 2. Summary of GO categories gene counts of the differentially expressed genes.

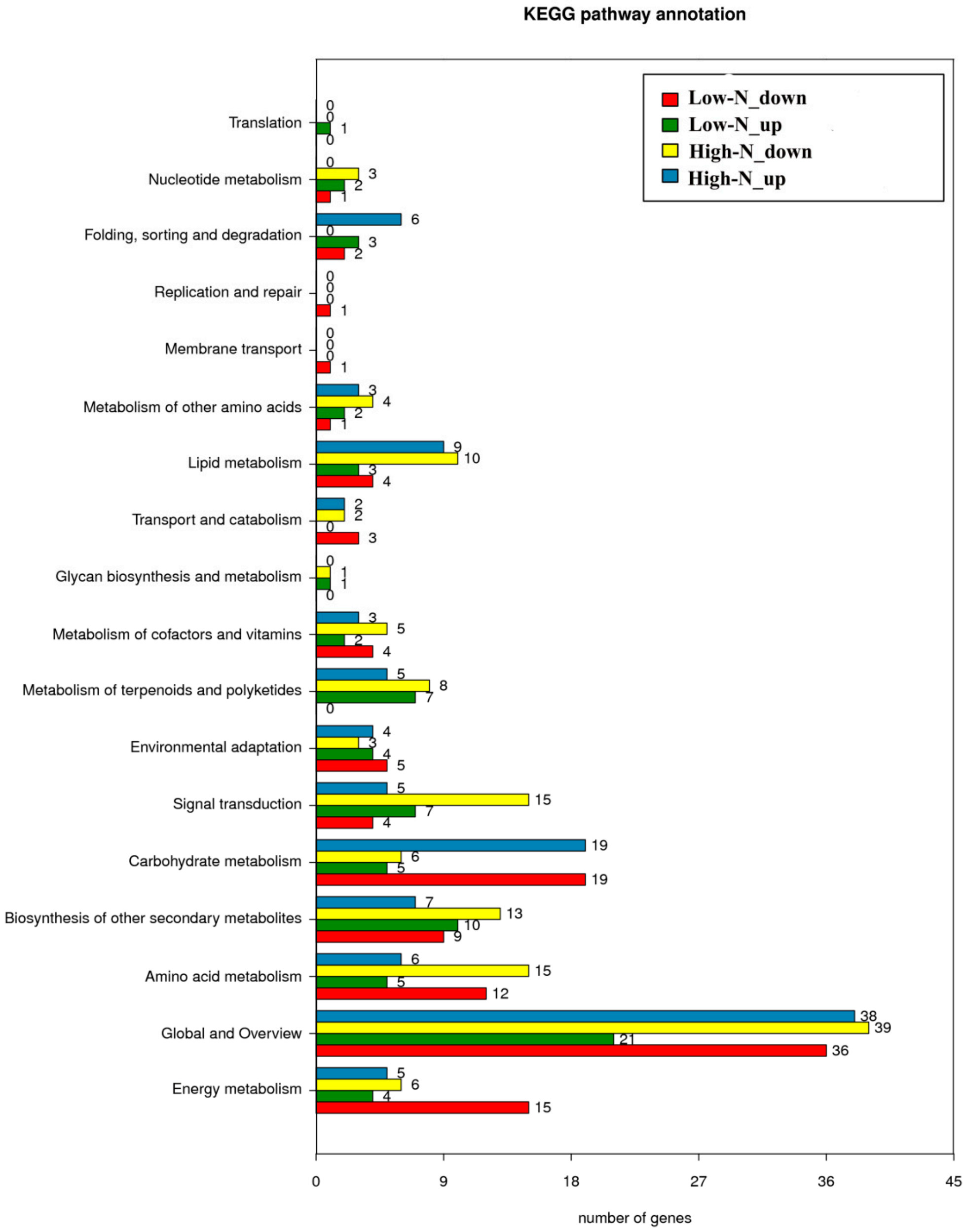

Supplementary Figure 3. Summary of KEGG pathway analysis of the differentially expressed genes.

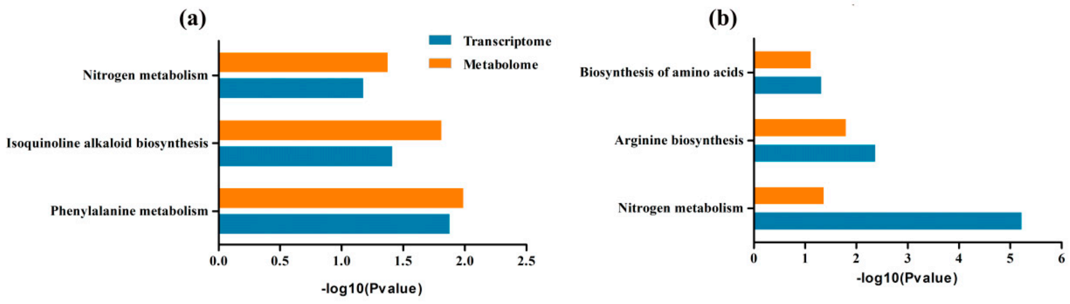

Supplementary Figure 4. The enrichment of KEGG pathway analysis of the differentially

expressed genes and changed metabolites under high (a) and low (b) nitrogen conduction.

Supplementary Table 1 Expression of TF genes under low nitrogen and high nitrogen.

Gene_ID	TF_ID	TF_Family	Low-N	High-N	Symbol
Os01g0705700	LOC_Os01g50940	bHLH	up		AIB
Os01g0952800	LOC_Os01g72370	bHLH	down		ORG2
Os03g0741100	LOC_Os03g53020	bHLH	up		BHLH92
Os04g0301500	LOC_Os04g23550	bHLH	up		BHLH35
Os08g0490000	LOC_Os08g38210	bHLH	up		BIM2
Os09g0475400	LOC_Os09g29930	bHLH	up		BIM2
Os10g0575000	LOC_Os10g42430	bHLH	up		MYC2
Os02g0120500	LOC_Os02g02820	bHLH		down	AMS
Os09g0455300	LOC_Os09g28210	bHLH		up	HEC2
Os03g0127500	LOC_Os03g03550	bZIP	up		RF2b
Os11g0154900	LOC_Os11g05640	bZIP	down		-
Os02g0728001	LOC_Os02g49560	bZIP		down	BZIP43
Os08g0176900	LOC_Os08g07970	bZIP		down	TGA4
Os03g0764100	LOC_Os03g55540	C2H2	up		-
Os05g0114400	LOC_Os05g02390	C2H2	up		ZAT12
Os05g0444200	LOC_Os05g37190	C2H2	up		WIP3
Os09g0431900	LOC_Os09g26210	C2H2	up		ZFP2
Os07g0667300	LOC_Os07g47140	CO-like	up		COL13
Os02g0731700	LOC_Os02g49880	CO-like		up	COL16
Os01g0202500	LOC_Os01g10580	DBB	down		BBX22
Os04g0540200	LOC_Os04g45690	DBB	down		BBX21
Os06g0245900	LOC_Os06g13670	E2F/DP	down		E2FE
Os02g0739700	LOC_Os02g50630	E2F/DP		up	E2FE
Os08g0508700	LOC_Os08g39830	EIL		down	EIL3
Os02g0654700	LOC_Os02g43790	ERF	up		ERF1A
Os02g0656600	LOC_Os02g43940	ERF	up		ERF034
Os03g0182800	LOC_Os03g08460	ERF	up		ERF073
Os09g0287000	LOC_Os09g11480	ERF	down		ERF073
Os09g0522200	LOC_Os09g35030	ERF	up		DREB1A
Os02g0677300	LOC_Os02g45450	ERF		up	DREB1G
Os02g0325600	LOC_Os02g22020	G2-like	down	up	ARR1
Os07g0589200	LOC_Os07g40020	GRAS	up		SCL32
Os05g0500600	LOC_Os05g42130	GRAS		up	SCL32
Os02g0149900	LOC_Os02g05640	HD-ZIP	down		HOX18
Os02g0649300	LOC_Os02g43330	HD-ZIP	up		HOX24
Os02g0232000	LOC_Os02g13800	HSF	up		HSFC2A
Os08g0159500	LOC_Os08g06280	LSD	down		LSD1
Os08g0494100	LOC_Os08g38590	M-type_MADS		up	MADS21
Os05g0140100	LOC_Os05g04820	MYB	up		MYB86

Os01g0663051	LOC_Os01g47370	MYB_related	up	down	RL3
Os02g0685200	LOC_Os02g46030	MYB_related	up		RVE1
Os05g0567600	LOC_Os05g49240	MYB_related	up	down	RL3
Os11g0700500	LOC_Os11g47460	MYB_related	down	down	MYBAS1
Os01g0975300	LOC_Os01g74410	MYB_related		down	MYB59
Os05g0579700	LOC_Os05g50350	MYB_related		down	RL6
Os12g0567300	LOC_Os12g37970	MYB_related		down	MYBAS2
Os01g0675800	LOC_Os01g48446	NAC	up		NAC68
Os01g0884300	LOC_Os01g66120	NAC	up		NAC48
Os06g0675600	LOC_Os06g46270	NAC	up		NAC021
Os07g0225300	LOC_Os07g12340	NAC	up	down	NAC67
Os07g0684800	LOC_Os07g48550	NAC	up		NAC100
Os08g0436700	LOC_Os08g33910	NAC	down		FEZ
Os03g0109000	LOC_Os03g01870	NAC		down	NAC021
Os03g0327800	LOC_Os03g21060	NAC		down	ONAC010
Os12g0618600	LOC_Os12g42400	NF-YA	up		NFYA10
Os03g0251350	LOC_Os03g14669	NF-YC	down	up	NFYC4
Os02g0136000	LOC_Os02g04340	Nin-like	up		NLP6
Os09g0549450	LOC_Os09g37710	Nin-like	down		NLP1
Os06g0145800	LOC_Os06g05350	Whirly	down		WHY1
Os01g0821600	LOC_Os01g60640	WRKY	up		WRKY46
Os01g0826400	LOC_Os01g61080	WRKY	up		WRKY33
Os08g0386200	LOC_Os08g29660	WRKY	up		WRKY53
Os09g0417600	LOC_Os09g25060	WRKY	up		WRKY40
Os11g0117400	LOC_Os11g02520	WRKY	up		WRKY46
Os11g0117500	LOC_Os11g02530	WRKY	up		WRKY70

Supplementary Table 2 Expression of phytohormones signal transduction related genes under low nitrogen and high nitrogen.

**Gene_ID**	**Low-N**	**High-N**	**Symbol**
Os01g0859300	up		*OsABI5*
Os01g0948900		up	*OsNPR5*
Os03g0180900	down		*OsJAZ2*
Os03g0181100	down		*OsJAZ4*
Os03g0268600	down	up	*OsPP2C30*
Os03g0402800	down		*OsJAZ5*
Os03g0860100	down		*OsERF83*
Os04g0442000	down		*OsARF9*
Os04g0456900		up	*OsEIL6*
Os04g0673300	down		*OsRR6*
Os05g0457200	down		*OsPP2C49*
Os05g0586200	down		*OsJAR1*
Os06g0562200	up		*OsPYL2*
Os07g0128800		up	-

Os07g0129200	up	down	*OsPR1a*
Os07g0129300	up	down	*OsPR1*
Os07g0243800		down	-
Os07g0592600	down		*OsGH3-8*
Os09g0325700		up	*OsPP2C1*
Os10g0191300	up	down	*OsPR1-101*
Os10g0391400	down	up	*OsJAZ13*
Os10g0392400	down		*OsJAZ1*

Supplementary Table 3 Sequences of primers used in this study.

Gene name	Forward primer	Reverse prime
Os01g0355250	TCAGGACATCAGTGTGCCAC	CCTTCACACGCTCTGTAGGG
Os01g0865100	GAAGCACGTCACAGAGGTCA	TTCGAACCCGGACTGAGTTG
Os02g0620500	AGGGAGTACGTCGAGCTGAT	CCTGTGCAGCACGTAGAAGA
Os03g0183600	GGCTGTCAACAAAGCACCTG	GTATCTTCTCCTCCTGCGGC
Os03g0223400	AACCTCAACCTCAGCGACTG	GTAGTTCCACTTCGGCAGCT
Os03g0291500	CCTGATAGCTGCACGAGAGG	CGTACGTCTCGTCGTGGTAG
Os03g0432100	TCCTCAACCTCGGCCTCAA	TCCATAACGACGTTGCCGAA
Os03g0663400	CGACCAAGGACACGGAGTTC	GACGATCTGGTAGTCGGTGC
Os04g0664900	GGACTTCTACATGGTGGGGC	TGCTGTCGTACTCGTTGACC
Os04g0680400	GGGATTACCGGAGCTTCCTG	TGCTGCTCTTGTACCAGTGG
Os05g0247100	GGCACCTACAAGCTGGACAT	AGGTAGTCCTCGAGGTCGAC
Os06g0169001	GGTGGAAATGGAGGAGGTCG	GTTCCTTCCCATCTGTGCCA
Os07g0104500	CAACATGAGCAAGATCGGCG	GAGGAGGAAGAGAAGCGAGC
Os07g0115300	GTTCGCACACTGCAACACAT	CATCCCGTTCTGCAGGTTCT
Os07g0129300	GGAGAAGCAGTGGTACGACC	GGCGAGTAGTTGCAGGTGAT
Os08g0424100	GGAGGTACGACATGGAGCTG	TTGGATCCACCCCACCATTG
Os09g0497900	TCTCAACAAGTGCGAGCCAT	CTAACTCCGGTGTTGCTGGT
Os09g0506000	AAAGCTCCGGTTTACCCTCG	CAACAGTACGGGCAGGTTCT
Os10g0191300	GGACTACGTCAACCTGCACA	CCAGAAGATGTTCTCGCCGT
Os10g0390500	CCCTCAGCTTAGTCTGCCAC	ATCTGATGTGCCATGTGCCA
Os10g0471300	GCCCGCCATCTACAGATTGA	TGACCACCACGCGATCTTTT
Os10g0530900	TCGTGCAGTACATCGACGAG	GACCTTGTCGTCGACGTAGG
Os10g0538200	TTCGTCAACATCTCCGGCAA	CTTCTTGACGGCCTGGTAGG
Os10g0561800	ACACCCTCAATCCCAAGCAG	GTCTGCTTCAGCTTCGTCCT
Os10g0580400	GCACCTACGACATCTACCGG	ACAATGACTCCCATGGCCAG
Os11g0679000	ATCATCTCGTGACGTGGTCG	TCCTTGGCCATCTGCTTCTG
Os12g0555100	GGAAGGTCTTCTCGAACCCG	CCTCCAGCACCTCTGACTTG
